# The Impact of Integration of a Genetic Clinic Into a Pediatric Cardiac Unit

**DOI:** 10.7759/cureus.50941

**Published:** 2023-12-22

**Authors:** Ayman Elfky, Yasser A Bhat, Abdulrahman Almesned, Abdullah Alqwaee, Ali Al-Akhfash, Zuhair Alhassnan

**Affiliations:** 1 Pediatric Cardiology, Prince Sultan Cardiac Center, Al Hasa, SAU; 2 Pediatric Cardiology, Prince Sultan Cardiac Center, Buraidah, SAU; 3 Medical Genetics, King Faisal Specialist Hospital and Research Centre, Riyadh, SAU

**Keywords:** pediatric cardiology, genetic counseling, genetic tests, congenital heart disease, cardiomyopathy, genetic clinic

## Abstract

Background: Previously published studies suggest that genetic or environmental causes can be observed in 20-30% of congenital heart disease (CHD) patients, which include aneuploidy, single gene defects, pathological copy number variations, and de novo autosomal dominant and recessive inheritance. Moreover, the genetic background of childhood cardiomyopathies (CMs) has not been elucidated well.

Objective: The study highlights the value of genetic assessment in diagnosing and family counseling for CHD and pediatric CM patients referred to the genetic clinic in a pediatric cardiology department.

Methods: The study involved patients less than 18 years of age attending the cardiogenetic clinic in the pediatric cardiology department between December 2010 and February 2019. The following patient categories who had genetic evaluation were included: CHD in the presence of a syndromic phenotype, patients with CHD having extracardiac congenital anomalies or delayed development, hypertrophic and dilated CM patients, patients with dilated aortic root and ascending aorta, significant CHD in siblings or first-degree relatives, suspected channelopathies; and interrupted aortic arch abnormalities.

Results: A total of 285 patients were evaluated in the cardiogenetic clinic. The mean age was 20.2 months, with a range of 0-168. Females and males constituted 153 (53.7%) and 132 (46.3%), respectively. The most common cause of referral to the genetic clinic was the presence of CM (N=134 (46.3%)): hypertrophic CM in 24% and dilated CM in 20% of cases. Seventy-six patients (26.7%) had positive genetic results. The most common genetic abnormality was familial infantile hypertrophic CM-causing gene *ELAC2* in 19 (23.5%) cases.

Conclusion: It may be beneficial for any pediatric cardiology unit to provide an established genetic clinic. Using a genetic clinic will enhance understanding of CHD pathophysiology, family education, and genetic counseling. Agreement on a well-written protocol and the way forward to specify what congenital heart conditions require genetic investigation should be clarified.

## Introduction

Epidemiological studies have suggested that a genetic or environmental cause can be identified in 20-30% of congenital heart disease (CHD) cases. Disorders of a single gene are found in 3-5%, and chromosomal defects/general aneuploidy in 8-10% of cases. Moreover, pathogenic copy number variations (CNVs) are observed in 3-25% of syndromic CHD cases and 3-10% of isolated CHD cases. De novo autosomal dominant variations are attributed to 8% of cases, and inherited autosomal recessive variations are attributed to 2%. Environmental causes can be identified for 2% of CHD cases. The remaining congenital HD is assumed to be multifactorial [1]. The list of genes associated with CHD is rapidly expanding. Maternal factors that might increase the risk of having a child with CHD include pre-gestational diabetes, phenylketonuria, febrile illnesses, rubella infection, upper respiratory tract viral infections, alcohol consumption, cigarette smoking, and exposure to teratogenic agents [2,3]. The genetic background of childhood cardiomyopathies (CMs) has not been studied well so far, and it is recommended to have a genetic evaluation for all types of CM patients and at-risk family members [4,5].

For clinicians caring for a child with CHD, it is important to determine if there is an underlying genetic component for several reasons: other important organ systems may be involved, there can be predictive information about clinical outcomes, and other family members may benefit from genetic screening [6]. The first step is to create a detailed family pedigree with a detailed phenotypic description to identify potentially inherited defects and to guide further genetic investigation. Genetic investigation into CHD may potentially predict the recurrence risk, define the pattern of inheritance within the family, help in family counseling, and evaluate the need for further family screening. In some circumstances, prenatal or pre-implantation genetic screening could identify fetuses or embryos at high risk for CHD [7].

The use of genetic information to customize care for patients with CHD, risk stratification, prognosis, and counseling for families is steadily expanding as we learn more about the genetic contribution to CHD. Genetic screening is available to a growing proportion of families with CHD [2]. The study aims to describe the spectrum of genetic abnormalities in CHD and pediatric CM patients referred to the genetic clinic in a pediatric cardiology department. It also underscores the value of genetic evaluation of these patients for diagnosis and family counseling.

## Materials and methods

The study included patients less than 18 years of age attending the cardiogenetic clinic in the pediatric cardiology department of Prince Sultan Cardiac Center in the Al Qassim region of Saudi Arabia between December 2010 and February 2019, and the local Institutional Review Board approved the study (111869). The cardiogenetic clinic is held every three months and is supervised by a principal consulting geneticist. The genetic consultant evaluates the patients and requests specific genetic tests. Once the test results are available, patients are called back to the clinic for counseling regarding the prognosis of the genetic condition, inheritance, and management. Moreover, the geneticist advises about recurrence risk for subsequent pregnancies and provides information and referral on prenatal testing options.

Patient information extracted from the electronic medical records system and the medical records archive covered the data on demographics, clinical data, and family histories. Moreover, three-generation family pedigree information and genetic test results were recorded. The following patient categories who had genetic evaluation were included: CHD in the presence of a syndromic phenotype, patients with CHD having extracardiac congenital anomalies or delayed development, hypertrophic and dilated CM patients, patients with dilated aortic root and ascending aorta, significant CHD in siblings or first-degree relatives, suspected channelopathies, and interrupted aortic arch abnormalities. The following patient groups were excluded: trisomy 13, 18, and 21; patients who were syndromic or had congenital anomalies but had a normal cardiac evaluation; and patients with hypertrophic CM secondary to maternal diabetes.

Genetic evaluation

Based on the clinical findings and the family history, genetic testing was either targeted, such as fluorescence in situ hybridization (FISH) for 22q11 deletion and Williams syndrome, or a multi-gene panel, as used in patients with Noonan and long QT syndromes with aortopathy, extracardiac congenital anomalies, and CM. Furthermore, single nucleotide polymorphism-based chromosomal microarray and complete exome sequencing were performed in patients with broad genetic diagnoses without syndromic association (developmental delay with CHD) or if the targeted genetic tests were negative.

Statistical analysis

The data were stored and compiled in an Excel sheet (Microsoft Corporation, Redmond, Washington). Analysis was performed using IBM SPSS Statistics for Windows, Version 21 (Released 2012; IBM Corp., Armonk, New York).

## Results

A total of 285 patients were evaluated in the cardiogenetic clinic. The mean age was 20.2 months, range (0-168). Females and males constituted 153 (53.7%) and 132 (46.3%), respectively. The commonest cause of referral to the genetic clinic was the presence of CM (N=134 (46.3%)): hypertrophic CM in 24% and dilated CM in 20% of cases (Table 1).

**Table 1:** Indication for referral to the genetic clinic *Multiple congenital anomalies, Beckwith Weidman syndrome, Barth syndrome, chondroplasia punctata, Pompe disease, Crouzon syndrome, ONARY, LEOPARD syndrome, Turner syndrome, hereditary hemorrhagic telangiectasia, hypomelanosis of Ito, Klippel Feil syndrome, Ehler Danlos syndrome, Di George syndrome. ^#^Mucopolysaccharidosis, lysosomal storage disease, fatty acid oxidation defect, rickets ^†^Restrictive cardiomyopathy. HCM, hypertrophic cardiomyopathy; DCM, dilated cardiomyopathy; CHD, congenital heart disease.

Diagnosis	Number (N)	Percentage (%)
HCM	69	24.2
DCM	57	20.0
CHD with associated congenital anomalies	49	17.2
CHD with a family history of CHD	18	6.4
Dysmorphic features*	16	5.5
Features of an identifiable syndrome*	15	5.2
Aortic root dilatation	11	3.9
William syndrome	10	3.5
Long QT	9	3.2
Marfan syndrome	8	2.8
Noonan syndrome	7	2.5
Metabolic disorder^#^	5	1.8
CHD with developmental delay	4	1.4
Connective tissue disorders	3	1.1
Duchene muscular dystrophy	2	0.7
Others^†^	2	0.7
Total	285	100

Of the 285 cases seen at the genetic clinic, 76 patients (26.7%) had positive genetic results. The most common genetic abnormality was familial infantile hypertrophic CM caused by the gene ELAC2 in 19 (23.5%) cases, followed by very long-chain acyl-CoA dehydrogenase deficiency gene defect (VLCAD) in 17 (21%) cases (Table 2).

**Table 2:** Genetic test results of the patients referred to the genetic clinic ELAC2, familial infantile hypertrophic cardiomyopathy; VLCAD, very long-chain acyl-CoA dehydrogenase; EFEMP2; familial aortic root aneurysm; 7q11.23, William's syndrome; KCNH2, long QT syndrome; NEXN, familial dilated cardiomyopathy; GAA, Pompe's disease; FBN1, Marfan syndrome; PTPN11, Noonan syndrome; ALMS1, Alstrom syndrome; MPS, mucopolysaccharidosis; DMD, Duchene muscular dystrophy; ACAD9, acyl Co-A dehydrogenase; CHR, chromosome; ENG, hereditary hemorrhagic telangiectasia; SLC22A5, carnitine deficiency; CHILD, congenital hemidysplasia with ichthyosiform erythroderma and limb defects.

Gene abnormality	N	%
ELAC2	19	23.5
VLCAD deficiency	17	21.0
EFEMP2	9	11.1
7q11.23	5	6.2
KCNH2	4	4.9
NEXN	4	4.9
GAA	4	5.0
FBN1	3	3.7
PTPN11	3	3.7
ALMS1	3	3.7
MPS	3	3.7
DMD	2	2.5
ACAD9	1	1.2
Duplication/deletion CHR2q/CHR8p	1	1.2
ENG	1	1.2
SLC22A5	1	1.2
CHILD Syndrome	1	1.2
Total	81	100

## Discussion

A significant proportion of CHD and CM patients require genetic evaluation, especially in our population with higher consanguinity rates. The study shows that 27% of the patients had a genetic abnormality, indicating a need for reliable and robust genetic services catering to pediatric CHD and CM patients. The CM patients formed the bulk of the referrals, and ELAC2 gene abnormality resulted in fatal infantile hypertrophic CM. These patients usually present in early infancy with non-obstructive hypertrophic CM, depressed cardiac function, and pericardial effusion, and most die before their first birthday. The authors have previously published the outcome of patients with ELAC2 gene mutation [8]. Four patients with Pompe's disease had severe infantile non-obstructive hypertrophic CM. After the test results were positive for the GAA mutation, they were directed to the clinical geneticist's center to administer enzyme replacement therapy.

Two patients had an aortic root aneurysm and presented to the emergency room with moderate pericardial effusion. Both patients underwent emergency aortic root replacement elsewhere. Family screening showed multiple siblings with aortic root aneurysms and an abnormality in the EFEMP2 gene. Two of the authors published an autosomal recessive mutation of the EFEMP2 gene in nine children from four Saudi families with familial aortic root aneurysms [9]. These patients had no features of cutis laxa or any other collagen vascular condition.

Cardiovascular physicians should possess some basic knowledge about history taking, physical examination, and dysmorphology assessment and be able to recognize common syndromes. Moreover, physicians should know when to refer a CHD patient for genetic evaluation and understand the results of genetic tests [10]. Integrating a genetics clinic with a pediatric cardiology unit can help disseminate information and provide learning opportunities for fellows and related staff.

Knowing the genetic diagnosis in advance is important because it facilitates decision-making about whether cardiac surgery can be proposed. Currently, our center does not offer surgical repair of CHDs for trisomies 13 and 18. Recent studies have shown that patients with genetic abnormalities and CHD are more likely to have postoperative complications [11,12]; therefore, knowing the genetic diagnosis before surgery is essential to counsel families and anticipate postoperative complications.

In cases of a known genetic abnormality associated with CHD or CM, the utilization of in vitro fertilization and selection of healthy gametes may help families wishing to have healthy children. Three families at our clinic have a history of multiple sibling deaths from CM. They later had normal children through a prenatal gamete selection program based on abnormal genetic identification.

A genetic clinic aims to deliver personalized care and optimal management of patients and their families. The consensus is that genetic services should be provided in a multidisciplinary clinic to achieve the best outcomes for patients and their families [13]. The study shows the importance of integrating a genetic clinic into the pediatric cardiac unit. Twenty-seven percent of referrals had a confirmed genetic defect; however, the study did not include trisomies 13 and 18, which would have raised the number of positive cases. A similar study on adult cardiac patients showed a yield of 47.6% [14]. Since not all patients in the cardiology clinic are screened, the figure may be higher than described in the study. As the burden of cardiovascular disease continues to grow, a better understanding of the underlying genetics is essential for improved care of patients with CHD [15].

Limitation

The study focuses on the pattern of genetic abnormalities diagnosed in a pediatric cardiology outpatient department and does not detail the studied population's clinical presentation and outcome measures. It also does not measure family satisfaction and patient care improvement parameters concerning the genetic clinic.

## Conclusions

From chromosomal aneuploidy to single gene defects, genetic abnormalities have a much greater influence on the development of various types of CHDs and CMs than previously appreciated. Providing a well-established genetic clinic may be beneficial for any pediatric cardiology service. Agreement for a well-written protocol and pathway to specify which CHDs require genetic investigation should be clarified. Utilizing a genetic clinic will improve the understanding of the pathophysiology of CHD, family education, and genetic counseling.
